# Supplementary material for: Identification of a mimotope of a complex gp41 human immunodeficiency virus epitope related to a non-structural protein of Hepacivirus previously implicated in Kawasaki disease
Source: Microbiol Spectr. 2025 Mar 31;13(5):e01911-24. doi: 10.1128/spectrum.01911-24 (PMC12054109; doi:10.1128/spectrum.01911-24)
Supplement: Supplemental File S1 — TMOD1 alignment with peptides identified from this study Blast and CoBalt were used to show potential acidic rich regions that may account for cross-reactivity. [file spectrum.01911-24-s0001.pdf]

```
Peptide 1- Arachis hypogaeya allergen                                        ADEEEEYDEDEYEYDEEDR
Peptide 2- Hepacivirus non-structural protein                                  VIPDREVLYQEFDEMEE
TMOD1 (refseq NM_003275)                               1 MSYRRELEKYRDLDEDKILGALTEEELRTLENELDELDPDNALLPAGLRQKDQTTKAPTGPFKREELLDHLEKQAKEFKD 80

MEME-derived (Similar to KD4-2H4/Hepacivirus related)                                KPAVIPDREALYQDIDEMEEC
TMOD1 (refseq NM_003275)                              81 REDLVPYTGEKRGKVWVPKQKPLDPVLESVTLEPELEEALANASDAELCDIAAILGMHTLMSNQQYYQALSSSSIMNKEG 160


TMOD1 (refseq NM_003275)                             161 LNSVIKPTQYKPVPDEEPNSTDVEETLERIKNNDPKLEEVNLNNIRNIPIPTLKAYAEALKENSYVKKFSIVGTRSNDPV 240

Peptide 3- Homo sapiens coagulation factor 8                                            NEEAEDYDDDLTDSEMD
TMOD1 (refseq NM_003275)                             241 AYALAEMLKENKVLKTLNVESNFISGAGILRLVEALPYNTSLVEMKIDNQSQPLGNKVEMEIVSMLEKNATLLKFGYHFT  320


TMOD1 (refseq NM_003275)                             321 QQGPRLRASNAMMNNNDLVRKRRLADLTGPIIPKCRSGV  359
```

**Supplemental Figure 1: TMOD1 alignment with peptides identified from this study**
Blast and CoBalt were used to show potential acidic rich regions that may account for cross-reactivity.
https://blast.ncbi.nlm.nih.gov/Blast.cgi
