# Supplementary material for: Identification of a mimotope of a complex gp41 human immunodeficiency virus epitope related to a non-structural protein of Hepacivirus previously implicated in Kawasaki disease
Source: Microbiol Spectr. 2025 Mar 31;13(5):e01911-24. doi: 10.1128/spectrum.01911-24 (PMC12054109; doi:10.1128/spectrum.01911-24)
Supplement: Table S1 — Notable peptides from the screen with 76Canc. [file spectrum.01911-24-s0002.pdf]

Supplemental Table 1: Notable peptides from the screen with 76Canc. Results from screen of PEPperCHIP® Human Epitome Microarray (PEP20195031237). Top binders, notable controls, HIV-related, SARS-related, and all peptides with FDEM motif are listed. Peptides are shaded to denote resolved meme (Blue for meme 1 and 2, green for meme 3, and mustard for the *Arachis Hypogaea* peptides that did not resolve a meme). HIV peptides that overlapped with the 76C group E657 motif are denoted by red text. Relationship to **Figure 2** and **Figure 3** from manuscript are denoted in the second and third column. Normalized binding units are green shaded in the fourth column.

Peptide	Related to resolved Meme (Figure 2)	Peptide group (Figure 3)	5ug/mL Corrected binding	*Organism*	Protein	IED link
DEEEEYDEDEYEYDE	no	1	2,444.0	*Arachis Hypogaea*	Glycinin	7959
EEEEYDEDEYEYDEE	no	1	2,018.5	*Arachis Hypogaea*	Glycinin	99180
RADEEEEYDEDEYEY	no	1	1,986.0	*Arachis Hypogaea*	Glycinin	99596
EEYDEDEYEYDEEDR	no	1	1,751.0	*Arachis Hypogaea*	Glycinin	174088
YVRQLEQYFDNFDQFL	3	5	1,173.0	*Plasmodium Vivax Sal-1*	Vacuolar Atp Synthase Catalytic Subunit A, Putative	66205
FLEDVPWLEDVDFLED			974.5	*Homo Sapiens*	Cerebellar Degeneration-Related Antigen 1	130831
CDKNTGDYYEDSYED			865.0	*Homo Sapiens*	Coagulation Factor Viii Precursor	134015
NEEAEDYDDDLTDSEMD		3	789.0	*Homo Sapiens*	Coagulation Factor Viii Precursor	129121
VDHFADGYDE		4	774.0	*Aspergillus Fumigatus*	Major Allergen Asp F 2 Precursor	106948
PVNDLCYPGDFNDYEEL			771.5	*Influenza A Virus (A/Viet Nam/1203/2004(H5N1))*	Hemagglutinin	97267
SFSKYVRQLEQYFDNFD	3		689.0	*Plasmodium Vivax Sal-1*	Vacuolar Atp Synthase Catalytic Subunit A, Putative	66205
EYDEDEYEYDEEDRR	no	1	602.0	*Arachis Hypogaea*	Glycinin	99235
DAWREGEEFVVEFDL			577.5	*Mycobacterium Leprae*	18 Kda Antigen	7680
EDYDDDLTDSEMDVVRF			566.0	*Homo Sapiens*	Coagulation Factor Viii Precursor	129121
LSFSCYLSVTEQSEFYF			537.0	*Human Hepatitis A Virus Hu/Australia/Hm175/1976*	Genome Polyprotein	39352
KNNEEAEDYDDDLTD			471.5	*Homo Sapiens*	Coagulation Factor Viii Precursor	129121
DSEEEDDEEEDDEDE			459.5	*Homo Sapiens*	Major Centromere Autoantigen B	170643
VIPDREVLYQEFDEMEE	1, 2	2	356.0	*Hepatitis C Virus Subtype 1A*	Polyprotein	46991
WVDHFADGYD			338.0	*Aspergillus Fumigatus*	Major Allergen Asp F 2 Precursor	106999
LQSDQEEIDYDDTISVE			335.0	*Homo Sapiens*	Coagulation Factor Viii Precursor	140019
TTYEEFSAKLDRLDEEF			334.0	*Leishmania Donovani*	Kinetoplastid Membrane Protein-11 - Leishmania Donovani	41164
KVSLLEIYNEELFDL			328.5	*Homo Sapiens*	Kinesin-Like Protein Kif11	171979
VPDRELLYQEFDEMEEC	1, 2	2	328.0	*Hepatitis C Virus*	Hepatitis C Virus Core, Matrix, Envelope And Non-Structural Protein Rna.	46834
IPDRELLYREFDEMEEC	1, 2	2	317.5	*Hepatitis C Virus*	Nonstructural Protein	46989
ALSSQHQAZIEIESFYE	3		313.0	*Homo Sapiens*	78 Kda Glucose-Regulated Protein	167209
QATGFGTNETEYLFE			311.5	*Ebola Virus - Mayinga, Zaire, 1976*	Envelope Glycoprotein Precursor	50368
VPDREVLYQEFDEMEEC	1, 2	2	311.0	*Hepatitis C Virus Subtype 1A*	Nonstructural Protein	46982
NEEAEDYDDDLTDSE			311.0	*Homo Sapiens*	Coagulation Factor Viii Precursor	129121
REVLYQEFDEMEE			306.0	*Hepatitis C Virus (Isolate H)*	Genome Polyprotein	39374
IVIYGVDTGIDIGHADF			305.0	*Penicillium Chrysogenum*	Alkaline Serine Protease	62847
TMDYPARAHTFDDF			301.0	*Hepatitis E Virus (Strain Burma)*	Orf2	34905
QEFDEMEECASHLPYFE			286.0	*Hepatitis C Virus Subtype 1A*	Polyprotein	69884
IHRFYESGIVFEEYKDY			281.0	*Clostridium Botulinum*	Botulinum Neurotoxin Type B Precursor	26404
IPDREALYQEFDEMEEC	1, 2	2	276.5	*Hepatitis C Virus*	Nonstructural Protein	46823
GMNVNFGLKSEYDDSYD			274.0	*Haemophilus Influenzae Serotype B*	Outer Membrane Protein P1	78878
YFALEAYAFD			273.5	*Aspergillus Fumigatus*	Major Allergen Asp F 2 Precursor	107011
IPDREAVLYQEFDEMEE	1, 2	2	272.0	*Hepatitis C Virus Subtype 1B*	Genome Polyprotein	46971
IPDREALYREFDEMEEC	1, 2	2	269.5	*Hepatitis C Virus Subtype 1B*	Nonstructural Protein	46970
ALSSQHQARIEIESFYE	3		269.5	*Homo Sapiens*	78 Kda Glucose-Regulated Protein	167209
IPDREVLYQEFDEMEEC	1, 2	2	259.0	*Hepatitis C Virus (Isolate H)*	Genome Polyprotein	39374
SNPAATHQDIDFLIEEI			251.5	*Homo Sapiens*	Glutamate Decarboxylase 2	102216
GNIGIDVEDEDSDEDEF			248.5	*Homo Sapiens*	Calcium Channel, Alpha 1A Subunit Isoform 3	119908
TSFSKYVRQLEQYF	3		247.5	*Plasmodium Vivax Sal-1*	Vacuolar Atp Synthase Catalytic Subunit A, Putative	66205
EIIDQDKSDFVEEDE			242.0	*Gadus Morhua*	Parvalbumin Beta	189616
IDTDGDGFIDFNEFISF			242.0	*Phleum Pratense*	Chain A, Crystal Structure Of The Calcium-Binding Pollen Allergen Phl P 7	661
SFNLLSARLFGELEFWF		7	238.0	*Staphylococcus Aureus Subsp. Aureus Col*	Amino Acid Permease	15354
APDKEVLYEAFDEMEEC	1, 2	2	237.0	*Hepatitis C Virus Subtype 1B*	Polyprotein	5591
PQFLRWFEEWAEEFC			229.0	*Plasmodium Falciparum*	Erythrocyte Membrane Protein 1	236280
AEGEFRELLYEAFDDM			227.0	*Hepatitis C Virus*	HCV found in serum phage display	937
TISVEMKKEDFDIYDED			227.0	*Homo Sapiens*	Coagulation Factor Viii Precursor	140019
VPDREVLYQDFDEMEEC	1, 2	2	222.5	*Hepatitis C Virus*	Polyprotein	46835
YYVFFEEQEDEIIGF			219.0	*Rattus Norvegicus*	Thyroid Stimulating Hormone Receptor Precursor	122956
EFRALLFIPRZAPFDLF			216.5	*Homo Sapiens*	Heat Shock Protein 90Kd	240909
ISEYIEEVFLPDDCF			215.5	*Homo Sapiens*	Dna-Directed Rna Polymerase Iii Subunit Rpc1	171578
PGNRPTGWEEEEDVE			211.0	*Homo Sapiens*	Trinucleotide Repeat Containing 6A, Isoform Cra_B	132001
IPDREVLYREFDEMEEC	1, 2	2	210.5	*Hepatitis C Virus*	Genome Polyprotein	26622
RGQYLQLQELECDWEEV			210.0	*Homo Sapiens*	Envoplakin	122551
VPDREVLYREFDEMEEC	1, 2	2	208.5	*Hepatitis C Virus Subtype 1B*	Nonstructural Protein	46836
EYDQVVVGAEYD		6	208.0	*Neisseria Meningitidis*	Serotype 15 Outer Membrane Protein	81910
VRNSWDTNWGDNGYGYF			207.5	*Dermatophagoides Pteronyssinus*	Der P 1 Allergen Precursor	70793
NEEPIAPYHFDLSGHAF			207.0	*Phleum Pratense*	Pollen Allergen Phl P 1 Precursor	19398
LQTLKIRVHEGYEEFTM			204.0	*Influenza A Virus (A/Viet Nam/1203/2004(H5N1))*	Polymerase Protein Pb2	97293
EQGSENEEIFVEEVSA			204.0	*Plasmodium Falciparum*	Antigen 332, Ag332=Pf332 Gene Clone G1 Product	13834
WPQIWFDDEEDV			201.0	*Streptococcus pneumoniae*	670-6B polysaccharide	130636
IIPDREVLYREFDEMEE	1, 2	2	**200.0**	*Hepatitis C Virus*	Genome Polyprotein	26622
AYDKDRYTEEEREVYSY			197.0	*Streptococcus Dysgalactiae Subsp. Equisimilis*	Skc-2	47283
SQGISDDDNDSAVAEFF			197.0	*Human Hepatitis A Virus Hu/Australia/Hm175/1976*	Genome Polyprotein	60441
	**Control**					
APSVEESVAPSVEESVA		8	12.0	*Plasmodium Falciparum*	Liver Stage Antigen-3	68360
	**Other HIV**					
EQELLELDKWASLWNWF			42.5	*Human Immunodeficiency Virus 1*	Envelope Glycoprotein	179864
KWASLWNWFDITNWLWY			41.0	*Human Immunodeficiency Virus 1*	Envelope Glycoprotein	179864
KWASLWNWFNITNWLWY			31.0	*Human Immunodeficiency Virus 1*	Envelope Surface Glycoprotein Gp160, Precursor	236082
EKNEQELLELDKWASLW			31.0	*Human Immunodeficiency Virus 1*	Envelope Glycoprotein Gp160 Precursor	150180
WNWFDITNW			22.0	*Human Immunodeficiency Virus 1*	Envelope Glycoprotein	146625
ALDKWD			22.0	*Human Immunodeficiency Virus 1*	Envelope Glycoprotein	149255
LLELDKWA			20.0	*Human Immunodeficiency Virus 1*	Envelope Glycoprotein Gp160 Precursor	150180
NWFDIT			20.0	*Human Immunodeficiency Virus 1*	Envelope Glycoprotein	146624
LLELDKWASLW			18.0	*Human Immunodeficiency Virus 1*	Envelope Glycoprotein Gp160 Precursor	150180
LELDKWASL			14.5	*Human Immunodeficiency Virus 1*	Envelope Glycoprotein	146417
LELDKWAS			12.0	*Human Immunodeficiency Virus 1*	Envelope Glycoprotein	146417
RPQIDIQEMRIGPMAWY			11.5	*Hiv-1 O_Ant70*	Envelope Glycoprotein Gp160	119783
NEQELLELDKWASL			11.5	*Human Immunodeficiency Virus 1*	Envelope Glycoprotein Gp160 Precursor	150180
WNWFDITN			11.0	*Human Immunodeficiency Virus 1*	Envelope Glycoprotein	146624
NNTRKSIHLGPGRAFYA			10.0	*Hiv-1 M:A*	Envelope Glycoprotein	163040
ELDKWASLWNWFNITN			9.0	*Human Immunodeficiency Virus 1*	Envelope Surface Glycoprotein Gp160, Precursor	236082
TWNDTYGSNN			9.0	*Human Immunodeficiency Virus 1*	Envelope Glycoprotein	119657
NNNTRKSINIGPGRALY			8.0	*Human Immunodeficiency Virus Type 1 (Isolate Yu2)*	Envelope Glycoprotein Gp160 Precursor	187167
KSIHLGPGRAFYA			7.0	*Human Immunodeficiency Virus 1*	Envelope Glycoprotein	146401
ELDKWAG			6.0	*Human Immunodeficiency Virus 1*	Envelope Glycoprotein	149296
ELDHWAS			4.0	*Human Immunodeficiency Virus 1*	Envelope Glycoprotein	149295
NWFDITNWLWYIR			4.0	*Human Immunodeficiency Virus 1*	Envelope Glycoprotein	163068
WASLWNWFDITN			4.0	*Human Immunodeficiency Virus 1*	Envelope Glycoprotein	179864
CNRPGRGIKQIGPM			0.0	*Hiv-1 Group O*	Envelope Glycoprotein Gp160	119785
NRPGRGIKQIGPMSVYS			0.0	*Hiv-1 Group O*	Envelope Glycoprotein Gp160	119785
RGIKQIGPMSVYSGSLA			0.0	*Hiv-1 Group O*	Envelope Glycoprotein Gp160	119785
QIGPMSVYSGSLADLGG			0.0	*Hiv-1 Group O*	Envelope Glycoprotein Gp160	119785
MSVYSGSLADLGGNNWS			0.0	*Hiv-1 Group O*	Envelope Glycoprotein Gp160	119785
SGSLADLGGNNWSRI			0.0	*Hiv-1 Group O*	Envelope Glycoprotein Gp160	119785
KSIHLGPGRAFYATGDI			0.0	*Hiv-1 M:A*	Envelope Glycoprotein	163040
LGPGRAFYATGDIIG			0.0	*Hiv-1 M:A*	Envelope Glycoprotein	163040
TRKGIHIGPGRAFYAT			0.0	*Hiv-1 M:A*	Chain P, Crystal Structure Of Anti-Hiv-1 V3 Fab 3074 In Complex With A Vi	163687
GIHIGPGRAFYATGQIT			0.0	*Hiv-1 M:A*	Chain P, Crystal Structure Of Anti-Hiv-1 V3 Fab 3074 In Complex With A Vi	163687
GPGRAFYATGQITGD			0.0	*Hiv-1 M:A*	Chain P, Crystal Structure Of Anti-Hiv-1 V3 Fab 3074 In Complex With A Vi	163687
KGVRIGPGQA			0.0	*Hiv-1 M:A*	Envelope Glycoprotein	162656
TRKSIHLGPGRAFY			0.0	*Hiv-1 M:A*	Envelope Glycoprotein	163040
NYNKRKRIHIGPGRA			0.0	*Hiv-1 M:B_Mn*	Envelope Glycoprotein Gp160 Precursor	79327
NKRKRIHIGPGRAFYTT			0.0	*Hiv-1 M:B_Mn*	Envelope Glycoprotein Gp160 Precursor	79327
RIHIGPGRAFYTTKNII			0.0	*Hiv-1 M:B_Mn*	Envelope Glycoprotein Gp160 Precursor	79327
KRKRIHIGPGRAFYTTK			0.0	*Hiv-1 M:B_Mn*	Envelope Glycoprotein Gp160 Precursor	79327
RKRIHIGPGRAFYTTKN			0.0	*Hiv-1 M:B_Mn*	Envelope Glycoprotein Gp160 Precursor	79327
RKRIHIGPGRAFYTT			0.0	*Hiv-1 M:B_Mn*	Envelope Glycoprotein Gp160 Precursor	79327
KRIHIGPGRAFYTT			0.0	*Hiv-1 M:B_Mn*	Envelope Glycoprotein Gp160 Precursor	79327
NNTKKSIKIRPRQA			0.0	*Hiv-1 M:C*	Chain P, Crystal Structure Of Anti-Hiv-1 V3 Fab 3074 In Complex With A U	163039
NTKKSIKIRPRQAFYAT			0.0	*Hiv-1 M:C*	Chain P, Crystal Structure Of Anti-Hiv-1 V3 Fab 3074 In Complex With A U	163039
SIKIRPRQAFYATNGII			0.0	*Hiv-1 M:C*	Chain P, Crystal Structure Of Anti-Hiv-1 V3 Fab 3074 In Complex With A U	163039
RPRQAFYATNGIIG			0.0	*Hiv-1 M:C*	Chain P, Crystal Structure Of Anti-Hiv-1 V3 Fab 3074 In Complex With A U	163039
TRKSIRIGPGQAFY			0.0	*Hiv-1 M:C*	Envelope Glycoprotein	163689
RKRIRVGPGQTVY			0.0	*Hiv-1 M:C*	Env	163244

SIKIRPRQAFYA	0.0	*Hiv-1 M:C*	Chain P, Crystal Structure Of Anti-Hiv-1 V3 Fab 3074 In Complex With A U	163039
CERPQIDIQEMRIGP	0.0	*Hiv-1 O_Ant70*	Envelope Glycoprotein Gp160	119783
DIQEMRIGPMAWYSMGI	0.0	*Hiv-1 O_Ant70*	Envelope Glycoprotein Gp160	119783
MRIGPMAWYSMGIGGTA	0.0	*Hiv-1 O_Ant70*	Envelope Glycoprotein Gp160	119783
PMAWYSMGIGGTAGNSS	0.0	*Hiv-1 O_Ant70*	Envelope Glycoprotein Gp160	119783
YSMGIGGTAGNSSRA	0.0	*Hiv-1 O_Ant70*	Envelope Glycoprotein Gp160	119783
ELDKWA	0.0	*Human Immunodeficiency Virus*	Envelope Glycoprotein Gp160 Precursor	12988
LWNWFNITNWLWYIK	0.0	*Human Immunodeficiency Virus 1*	Envelope Surface Glycoprotein Gp160, Precursor	236082
LELDKWASLWNWFDITN	0.0	*Human Immunodeficiency Virus 1*	Envelope Glycoprotein	179864
LVCASRELERFAVNPGL	0.0	*Human Immunodeficiency Virus 1*	Gag Protein	165638
CASRELERFAVNPGLLE	0.0	*Human Immunodeficiency Virus 1*	Gag Protein	165638
ELRDKKQKVHALFYKLD	0.0	*Human Immunodeficiency Virus 1*	Envelope Glycoprotein	180966
RDKKQKVHALFYKLDIV	0.0	*Human Immunodeficiency Virus 1*	Envelope Glycoprotein	180966
YLKDQQLLGIWGCSGKL	0.0	*Human Immunodeficiency Virus 1*	Envelope Glycoprotein	94978
NEQELLELDKWASLWN	0.0	*Human Immunodeficiency Virus 1*	Envelope Glycoprotein	179863
SLWNWFDITNWLWYIK	0.0	*Human Immunodeficiency Virus 1*	Gp160	187194
GCSGKLICT	0.0	*Human Immunodeficiency Virus 1*	Envelope Glycoprotein	18911
ILKEPVHGV	0.0	*Human Immunodeficiency Virus 1*	Gag-Pol Polyprotein	27125
CTTAVPWN	0.0	*Human Immunodeficiency Virus 1*	Envelope Glycoprotein	7214
ELDKWKSL	0.0	*Human Immunodeficiency Virus 1*	Envelope Glycoprotein	149299
ELDKWNSL	0.0	*Human Immunodeficiency Virus 1*	Envelope Glycoprotein	149300
NEQELLELDKWASLW	0.0	*Human Immunodeficiency Virus 1*	Envelope Glycoprotein Gp160 Precursor	150180
ALDKWAS	0.0	*Human Immunodeficiency Virus 1*	Envelope Protein	149254
ALDKWQN	0.0	*Human Immunodeficiency Virus 1*	Envelope Glycoprotein	149256
ELDKWAN	0.0	*Human Immunodeficiency Virus 1*	Envelope Glycoprotein	149297
ELDKWAS	0.0	*Human Immunodeficiency Virus 1*	Envelope Glycoprotein	146417
ELEKWAS	0.0	*Human Immunodeficiency Virus 1*	Envelope Glycoprotein	149301
EQELLELDKWASLW	0.0	*Human Immunodeficiency Virus 1*	Envelope Glycoprotein Gp160 Precursor	150180
NWFDITNWLWYIK	0.0	*Human Immunodeficiency Virus 1*	Envelope Glycoprotein Gp160 Precursor	164136
NCTRPNNNTRKSINIG	0.0	*Human Immunodeficiency Virus Type 1 (Isolate Yu2)*	Envelope Glycoprotein Gp160 Precursor	187167
RPNNNTRKSINIGPGRA	0.0	*Human Immunodeficiency Virus Type 1 (Isolate Yu2)*	Envelope Glycoprotein Gp160 Precursor	187167
NTRKSINIGPGRALYTT	0.0	*Human Immunodeficiency Virus Type 1 (Isolate Yu2)*	Envelope Glycoprotein Gp160 Precursor	187167
TKKGIAIGPGRTLY	0.0	*Human Immunodeficiency Virus Type 1 (New York-5 Isolate)*	Truncated Surface Protein	163621
KSITIGPGRA	0.0	*Human Immunodeficiency Virus Type 1 (Sf162 Isolate)*	Envelope Glycoprotein Gp160 Precursor	150865
NWFDISKWLWYI	0.0	*Human Immunodeficiency Virus Type 1 (Sf162 Isolate)*	Envelope Glycoprotein Gp160 Precursor	150874

Other FDEM motif (<200)

IPDREVLYQGFDEMEEC	195.0	*Hepatitis C Virus*	Nonstructural Protein	46992
IPDREVLYQQFDEMEEC	192.0	*Hepatitis C Virus Subtype 1B*	Polyprotein	46993
VPDREILYQQFDEMEEC	183.0	*Hepatitis C Virus*	Genome Polyprotein	46998
REVLYREFDEME	164.0	*Hepatitis C Virus*	Genome Polyprotein	26622
PDREALYQEFDEMEECA	151.0	*Hepatitis C Virus*	Nonstructural Protein	47174
PAVIPDREVLYREFDEM	145.0	*Hepatitis C Virus*	Nonstructural Protein	46994
IPDREVLYQAFDEMEEC	143.0	*Hepatitis C Virus Subtype 1A*	Nonstructural Protein	46827
IPDREALYQQFDEMEEC	129.0	*Hepatitis C Virus Subtype 1B*	Precursor Protein	46824
PDRELLYREFDEMEECP	124.5	*Hepatitis C Virus*	Nonstructural Protein	47179
IPDREVLHREFDEMEEC	124.5	*Hepatitis C Virus Subtype 1B*	Nonstructural Protein	46826
LYQEFDEMEECSQHLPY	122.0	*Hepatitis C Virus (Isolate H)*	Genome Polyprotein	39374
TPDKEILYEAFDEMEEC	116.0	*Hepatitis C Virus Subtype 1B*	Polyprotein	71888
REVLYQEFDEMEECSQ	109.5	*Hepatitis C Virus (Isolate H)*	Genome Polyprotein	39374
PATVPDREVLYQEFDEM	107.5	*Hepatitis C Virus Subtype 1A*	Nonstructural Protein	46982
PDKEILYEAFDEMEECA	100.0	*Hepatitis C Virus Subtype 1B*	Polyprotein	47120
VPDREVLYREFDEMEAC	96.0	*Hepatitis C Virus Subtype 1B*	Precursor Polyprotein	47000
PDREVLYQDFDEMEECA	95.0	*Hepatitis C Virus*	Polyprotein	47182
PDREALYREFDEMEECA	92.0	*Hepatitis C Virus Subtype 1B*	Nonstructural Protein	47176
PDREVLYREFDEMEECA	88.5	*Hepatitis C Virus Subtype 1B*	Genome Polyprotein	47191
EFDEMEECSQHLPYIE	87.0	*Hepatitis C Virus (Isolate 1)*	Genome Polyprotein	11973
APDKEILYEAFDEMEEC	87.0	*Hepatitis C Virus Subtype 1B*	Polyprotein Precursor	71866
PDREVLYQEFDEMEECS	86.0	*Hepatitis C Virus (Isolate H)*	Genome Polyprotein	39374
FDEMEECSQHLPYIE	83.0	*Hepatitis C Virus (Isolate H)*	Genome Polyprotein	39374
PDREALYQQFDEMEECS	82.5	*Hepatitis C Virus Subtype 1B*	Precursor Protein	47175
VPDREVLYREFDEM	82.0	*Hepatitis C Virus Subtype 1B*	Nonstructural Protein	46836
PDKEVLYEAFDEMEECA	80.0	*Hepatitis C Virus Subtype 1B*	Polyprotein	47122
AVIPDREVLYQEFDEME	78.5	*Hepatitis C Virus Subtype 1A*	Polyprotein	46991
PDREILYQQFDEMEECS	78.0	*Hepatitis C Virus*	Genome Polyprotein	47177
PAVVPDREVLYQEFDEM	78.0	*Hepatitis C Virus Subtype 1B*	Polyprotein	46999
PAIVPDRELLYQEFDEM	73.0	*Hepatitis C Virus*	Hepatitis C Virus Core, Matrix, Envelope And Non-Structural Protein Rna.	46834
PAIIPDREVLYQEFDEM	73.0	*Hepatitis C Virus (Isolate H)*	Genome Polyprotein	39374
PATIPDREVLYREFDEM	73.0	*Hepatitis C Virus Subtype 1B*	Nonstructural Protein	46973
LYQEFDEMEECASHLPY	72.0	*Hepatitis C Virus Subtype 1B*	Polyprotein	58113
EALYREFDEMEECASHL	70.0	*Hepatitis C Virus Subtype 1B*	Nonstructural Protein	47176
PATIPDREVLYQEFDEM	69.0	*Hepatitis C Virus*	Nonstructural Protein	46972
PAVVPDREVLYREFDEM	67.5	*Hepatitis C Virus Subtype 1B*	Precursor Polyprotein	47000
EALYQEFDEMEECASHL	65.0	*Hepatitis C Virus*	Nonstructural Protein	47174
PAVIPDREVLYQEFDEM	64.0	*Hepatitis C Virus Subtype 1A*	Polyprotein	46991
EVLYREFDEMEECASHL	60.0	*Hepatitis C Virus Subtype 1B*	Polyprotein	47192
PATIPDREALYREFDEM	59.0	*Hepatitis C Virus Subtype 1B*	Nonstructural Protein	46970
QEFDEMEECASHLPYIE	58.0	*Hepatitis C Virus Subtype 1B*	Polyprotein	58113
PAIVPDREVLYQDFDEM	57.5	*Hepatitis C Virus*	Polyprotein	46835
PAIIPDREALYQEFDEM	57.0	*Hepatitis C Virus*	Nonstructural Protein	46823
PDREVLYQEFDEMEECG	56.0	*Hepatitis C Virus*	Polyprotein Precursor	47185
PAVIPDRELLYREFDEM	55.5	*Hepatitis C Virus*	Nonstructural Protein	46989
REFDEMEECSQHLPYIE	55.0	*Hepatitis C Virus Subtype 1A*	Hcv-1	40925
IIPDREVLYREFDEM	52.5	*Hepatitis C Virus*	Polyprotein	22246
QEFDEMEECSQHLPYIE	52.0	*Hepatitis C Virus (Isolate H)*	Genome Polyprotein	39374
EVLYQEFDEMEECASHL	52.0	*Hepatitis C Virus Subtype 1B*	Polyprotein	47183

SARS Spike related

CANLLLQYGSFCTQLN	31.5	*Sars Coronavirus*	Spike Glycoprotein Precursor	6011
LLLQYGSFCTQLNRALS	31.5	*Sars Coronavirus*	Spike Glycoprotein Precursor	6011
YGSFCTQLNRALSGIA	31.5	*Sars Coronavirus*	Spike Glycoprotein Precursor	6011
VNCTDVSTAIHADQLTP	23.0	*Sars Coronavirus*	Spike Glycoprotein Precursor	50531
QDVNCTDVSTAIHADQ	17.0	*Sars Coronavirus*	Spike Glycoprotein Precursor	50531
NCTDVSTAIHADQLTPA	17.0	*Sars Coronavirus*	Spike Glycoprotein Precursor	50531
FGRDVSDFTDSVRDPKT	5.0	*Sars Coronavirus*	Spike Glycoprotein Precursor	17524
KGIYQTSNFRVVPSGDV	2.0	*Sars Coronavirus*	Spike Glycoprotein Precursor	30988
YQTSNFRVVPSGDVVRF	2.0	*Sars Coronavirus*	Spike Glycoprotein Precursor	30988
ALNCYW	0.0	*Sars Coronavirus*	Spike Glycoprotein Precursor	2769
AMQMAYRF	0.0	*Sars Coronavirus*	Spike Glycoprotein Precursor	3176
ATEKSNVVRGWV	0.0	*Sars Coronavirus*	Spike Glycoprotein Precursor	4941
LQYGSFCTQLNRALSGI	0.0	*Sars Coronavirus*	Spike Glycoprotein Precursor	6011
GSFCTQLN	0.0	*Sars Coronavirus*	Spike Glycoprotein Precursor	6011
FGEVFNAT	0.0	*Sars Coronavirus*	Spike Glycoprotein Precursor	15972
FGRDVSDFTDSVRDPK	0.0	*Sars Coronavirus*	Spike Glycoprotein Precursor	17524
FQQFGRDVSDFTDSVRD	0.0	*Sars Coronavirus*	Spike Glycoprotein Precursor	17524
QFGRDVSDFTDSVRDPK	0.0	*Sars Coronavirus*	Spike Glycoprotein Precursor	17524
VSDFTDSV	0.0	*Sars Coronavirus*	Spike Glycoprotein Precursor	17524
HEGKAYFPREGV	0.0	*Sars Coronavirus*	Spike Glycoprotein Precursor	23685
NYNYKYRYLRHGKLRPF	0.0	*Sars Coronavirus*	Spike Glycoprotein Precursor	46681
PFSPDGKPCTPP	0.0	*Sars Coronavirus*	Spike Glycoprotein Precursor	47565
PLKPTKRSFIEDLLF	0.0	*Sars Coronavirus*	Spike Glycoprotein Precursor	48382
GRDVSDFTDSVRDPKTS	0.0	*Sars Coronavirus*	Spike Glycoprotein Precursor	49507
SDFTDSVRDPKTSE	0.0	*Sars Coronavirus*	Spike Glycoprotein Precursor	49507
RDVSDFTDSVRDPKTSE	0.0	*Sars Coronavirus*	Spike Glycoprotein Precursor	49507
DVSDFTDSVRDPKTSEI	0.0	*Sars Coronavirus*	Spike Glycoprotein Precursor	53435
SSEVAVLYQDVNCTDV	0.0	*Sars Coronavirus*	Spike Glycoprotein Precursor	60978
VITPGTNASSEVAVLY	0.0	*Sars Coronavirus*	Spike Glycoprotein Precursor	69129
LLFNKVTLADAGFMKQ	0.0	*Sars Coronavirus Bj01*	Spike Glycoprotein Precursor	37290
NKVTLADAGFMKQYGEC	0.0	*Sars Coronavirus Bj01*	Spike Glycoprotein Precursor	37290
LADAGFMKQYGECLGDI	0.0	*Sars Coronavirus Bj01*	Spike Glycoprotein Precursor	37290
ICHEGKAYFPREGVFVF	0.0	*Sars Coronavirus Bj01*	Spike Glycoprotein Precursor	46822
GVFVFNGTSWFITQRNF	0.0	*Sars Coronavirus Bj01*	Spike Glycoprotein Precursor	46822
FPREGVFVFNGTSWFIT	0.0	*Sars Coronavirus Bj01*	Spike Glycoprotein Precursor	46822
GKAYFPREGVFVFNGTS	0.0	*Sars Coronavirus Bj01*	Spike Glycoprotein Precursor	46822
PAICHEGKAYFPREG	0.0	*Sars Coronavirus Bj01*	Spike Glycoprotein Precursor	46822
KLRPFERDISNV	0.0	*Sars Coronavirus Bj01*	Spike Glycoprotein S	148529
